# Tumor-Directed Blockade of CD47 with Bispecific Antibodies Induces Adaptive Antitumor Immunity

**DOI:** 10.3390/antib7010003

**Published:** 2018-01-03

**Authors:** Elie Dheilly, Stefano Majocchi, Valéry Moine, Gérard Didelot, Lucile Broyer, Sébastien Calloud, Pauline Malinge, Laurence Chatel, Walter G. Ferlin, Marie H. Kosco-Vilbois, Nicolas Fischer, Krzysztof Masternak

**Affiliations:** Novimmune S.A., 14 chemin des Aulx, CH-1228 Geneva, Switzerland; elie.dheilly@epfl.ch (E.D.); smajocchi@novimmune.com (S.M.); vmoine@novimmune.com (V.M.); gdidelot@novimmune.com (G.D.); lbroyer@novimmune.com (L.B.); scalloud@novimmune.com (S.C.); pmalinge@novimmune.com (P.M.); lchatel@novimmune.com (L.C.); wferlin@novimmune.com (W.G.F.); mkosco-vilbois@novimmune.com (M.H.K.-V.); nfischer@novimmune.com (N.F.)

**Keywords:** bispecific antibodies, CD47, antitumor immunity, immunotherapy, immunosurveillance, checkpoint inhibitors, phagocytosis

## Abstract

CD47 serves as an anti-phagocytic receptor that is upregulated by cancer to promote immune escape. As such, CD47 is the focus of intense immuno-oncology drug development efforts. However, as CD47 is expressed ubiquitously, clinical development of conventional drugs, e.g., monoclonal antibodies, is confronted with patient safety issues and poor pharmacology due to the widespread CD47 “antigen sink”. A potential solution is tumor-directed blockade of CD47, which can be achieved with bispecific antibodies (biAbs). Using mouse CD47-blocking biAbs in a syngeneic tumor model allowed us to evaluate the efficacy of tumor-directed blockade of CD47 in the presence of the CD47 antigen sink and a functional adaptive immune system. We show here that CD47-targeting biAbs inhibited tumor growth in vivo, promoting durable antitumor responses and stimulating CD8^+^ T cell activation in vitro. In vivo efficacy of the biAbs could be further enhanced when combined with chemotherapy or PD-1/PD-L1 immune checkpoint blockade. We also show that selectivity and pharmacological properties of the biAb are dependent on the affinity of the anti-CD47 arm. Taken together, our study validates the approach to use CD47-blocking biAbs either as a monotherapy or part of a multi-drug approach to enhance antitumor immunity.

## 1. Introduction

CD47 is a ubiquitous anti-phagocytic receptor widely known as the “don’t eat me” signal, as it inhibits phagocytosis by engaging a signal regulatory protein alpha (SIRPα) on macrophages and other phagocytes (reviewed in [1,2,3,4]). In addition, CD47 carries out other important physiological functions, such as regulation of cardiovascular homeostasis, neuronal development, bone remodeling, adaptive immunity, cellular response to stress, stem cell renewal, cell adhesion, motility, proliferation, and survival (reviewed in: [1,2,4,5,6]).

Cancer cells upregulate CD47 expression in order to evade antitumor immunity. In nearly all hematological and solid cancers, increased levels of CD47 expression correlate with more aggressive disease and poorer prognosis (reviewed in [7,8,9]). Blockade of CD47 increases phagocytosis of tumor cells and stimulates macrophage-mediated tumor elimination in various human xenograft tumor models (reviewed in [7,8,9]). In addition, when studied in tumor models in immunocompetent animals, the induction of adaptive immune responses in mediating therapeutic efficacy of CD47 neutralization has been observed [10,11,12,13,14,15,16,17,18]. These studies revealed that CD47 expressed on tumor cells is a key suppressor of antitumor immunity as well as a mediator of resistance to PD1 checkpoint blockade therapy [19,20]. However, it is not clear how CD47 blockade facilitates tumor immunosurveillance. Several mechanisms have been proposed, including enhanced immune cell infiltration [11,15,19], dendritic cell activation [17,21], and increased tumor antigen cross-presentation with activation of cytotoxic T cell responses due to enhanced phagocytosis of tumor cells [7,8,22,23]. Given the involvement of CD47 in various physiological and cellular functions, multiple non-mutually exclusive mechanisms may be involved. As such, a rationale for therapeutically targeting CD47 in cancer has evolved. Thus, several CD47-directed therapeutic strategies are being pursued (reviewed in [8,9]).

Nonetheless, ubiquitous CD47 expression on healthy cells presents a considerable hurdle for the development of CD47-blocking therapies. Molecules that block CD47 have been observed to induce hematotoxicity in mice and non-human primates. Furthermore, they have poor pharmacokinetic properties, which reflects the substantial target-mediated drug disposition due to the CD47 antigen sink [16,24,25,26,27,28,29]. Hematotoxicity was found to be dependent on the Fc region of the molecules as CD47-blocking mAbs and SIRPα-Fc fusion proteins bearing a functional Fc region show significant hematological toxicity, while Fc effector functionless CD47 blockers display a better safety profile [16,26,29].

Dual targeting bispecific antibodies (biAbs) co-engage two antigens at the cell surface, which stabilizes binding through avidity [30,31]. Therefore, creating anti-CD47/antitumor-associated antigen (TAA) biAbs to steer CD47 blockade towards TAA-positive malignant cells, and away from TAA-negative healthy cells expressing CD47 alone, provides a solution to poor pharmacology and safety issues related to ubiquitous CD47 expression [32,33,34]. We have previously demonstrated that selective targeting of CD47 on tumor cells can be achieved with anti-CD47/TAA biAbs with a low-affinity anti-CD47 arm and a high-affinity anti-TAA arm [34]. Such an “unbalanced affinity” design minimizes CD47 binding to TAA-negative cells while driving bivalent attachment of the biAb to double-positive cells via TAA/CD47 co-engagement. Furthermore, with an increased selectivity, a biAb can be safely endowed with an immunologically active Fc region, thus maximizing tumor cell phagocytosis via the engagement of FcγRs and a concurrent blockade of the “don’t eat me” signal [34,35]. Indeed, the unbalanced affinity exemplified by our anti-human CD47/CD19 therapeutic biAb candidate, NI-1701, demonstrated potent antitumor activity in xenograft models. Moreover, in non-human primates, it displayed favorable pharmacokinetics and the absence of toxicity following multiple administrations of super-therapeutic doses [35].

However, the lack of cross-reactivity of anti-human therapeutic biAbs with mouse CD47 precludes in vivo evaluation in a physiologically relevant context, i.e., with tumors grown in syngeneic, immunocompetent mouse strains in the presence of the CD47 antigen sink. To overcome this limitation, we generated anti-mouse CD47-specific biAbs. Using these reagents and a syngeneic mouse model of B cell lymphoma, we show that the affinity of the anti-mouse CD47 biAb arm determines the safety, pharmacokinetic properties, and in vivo efficacy in the presence of the CD47 antigen sink. We observed that tumor-directed blockade of CD47 enhances tumor antigen cross-presentation and CD8^+^ T cell activation in vitro. Finally, we show that tumor-directed blockade of CD47 via biAbs can increase the therapeutic effect of standard of care oncology treatments such as PD-L1-blocking antibodies and cyclophosphamide.

## 2. Materials and Methods

### 2.1. Antibodies

Generation of bispecific antibodies based on one heavy chain and two different light chains (one κ and one λ), including light chain library construction, phage display selection and screening, bispecific antibody production, purification, and analytical characterization, are described in detail in [36]. Briefly, anti-mouse CD47 antibody sequences were identified with the κλ body phage display platform [36] using a combination of recombinant mouse CD47 (produced in-house) or cells naturally expressing or transfected with mouse CD47. Screening was performed as described in [36], and positive hits were reformatted into hIgG1 monoclonal antibodies for further evaluation. A panel of anti-mouse CD47 antibody arms with different affinities was generated and two of these candidates were used for the generation of bispecific antibodies analyzed in this study. The human anti-mouse CD47 variable domains (κ-light chain) and the human variable domain of an anti-human CD19 antibody (λ-light chain) [36] were grafted on mouse heavy and light chain constant domains, giving rise to bispecific human-mouse chimeric mIgG2a molecules (Figure S1). Therefore, the two mouse CD47-targeting biAbs (biAb1 and biAb2) comprised two distinct anti-mouse CD47 arms with different affinities (Table S1), a common, previously described, high-affinity antitumor associated antigen (TAA) antibody arm recognizing the human CD19 antigen (hCD19), and a wild type mIgG2a Fc portion. For the construction of monovalent anti-mouse CD47 antibodies, the anti-human CD19 arm was replaced with a non-binding arm (non-cross-reactive anti-human CD47 arm, ref. [34]). In addition, we generated human-mouse chimeric anti-CD47 mAbs, consisting of human anti-mouse CD47 variable domains of biAb1 and biAb2 grafted on mouse IgG2aλ constant domains (CD47 mAb1 and CD47 mAb2). Finally, the human anti-human CD19 variable domain was also used to generate a chimeric CD19 mAb and a chimeric CD19 monovalent antibody, both with a mouse IgG2a constant region.

### 2.2. Cell Lines and Reagents

Mouse Diffuse Large B Cell Lymphoma cell line A20 (ATCC (Wesel, Germany) TIB208™, BALB/c) was cultured in RPMI-1840 medium and supplemented with 10% heat-inactivated fetal calf serum (FCS), 2 mM l-glutamine, 1 mM sodium pyruvate, 10 mM HEPES, 5 mM d-glucose (all from Sigma-Aldrich, Buchs, Switzerland) and 50 μM 2-mercaptoethanol (Gibco/Thermo Fisher Scientific, Reinach, Switzerland). Mouse melanoma cell line B16–F10 (ATCC^®^ CRL6475™, C57Bl/6) was cultured in DMEM-Hi glucose medium and supplemented with 10% heat-inactivated FCS, 4 mM l-glutamine, and 1 mM sodium pyruvate. Cells were maintained at 37 °C in a water-jacketed incubator containing 5% CO_2_. A20-hCD19, A20-hCD19-HA-GFP, A20-mCherry, A20-hCD19-GFP, and B16F10-hCD19 cell lines were obtained by transfecting with Lipofectamine 2000 (Thermo Fisher Scientific, Reinach, Switzerland) the parental wild type cell lines with the corresponding transgenes: human CD19 transmembrane and extracellular domains (UniProtKB-P15391), and hemagglutinin (HA) gene from influenza virus strain A/Puerto Rico/8/1934 H1N1 (UniProtKB-P03452). Proteins were addressed to the cell surface via a signal peptide. Stably expressing pools were enriched by successive Fluorescence-Activated Cell Sorting (FACS) of positive cells. Once stable pools were obtained, clones were generated by single cell FACS sorting in the absence of selective pressure. Cell surface mCD47 and hCD19 densities were determined by an indirect immunofluorescence assay (QIFIKIT) according to the manufacturer’s instructions (Agilent Technologies, Basel, Switzerland). As the primary antibody, saturating concentrations of the high-affinity anti-mCD47 mAb1 and the anti-hCD19 mAb described in this manuscript were used.

### 2.3. Mice

Six- to eight-week-old female BALB/c mice and C57BL/6J mice were purchased from Charles River Laboratories (Ecully, France). All animal experiments were performed in accordance with the Swiss Federal Veterinary Office guidelines and as authorized by the Cantonal Veterinary Office.

### 2.4. Antibody Binding Assays

Harvested cells were washed with a FACS buffer (PBS, 2% BSA, and 0.1% sodium azide), incubated with test antibodies for 30 min at 4 °C in FACS buffer, washed once to remove unbound antibody, and stained with a fluorescently labeled anti-mouse IgG detection antibody (either an anti-mouse IgG (H + L) (A865, Life technologies/Thermo Fisher Scientific, Reinach, Switzerland) or an anti-mouse IgG (Fc) (115-606-072 Jackson ImmunoResearch, West Grove, PA, USA) for 15 min at 4 °C. A viability marker, SYTOX blue (Thermo Fischer Scientific, Reinach, Switzerland) or propidium iodide (Sigma-Aldrich, Buchs, Switzerland), was added before acquisition to exclude dead cells. Antibody binding was measured by flow cytometry using either a Cytoflex flow cytometer (Beckman Coulter, Indianapolis, IN, USA) or a FACS Calibur flow cytometer (BD Biosciences, San Jose, CA, USA). FACS data were analyzed with FlowJo software (FlowJo LLC, Ashland, OR, USA).

For binding assays performed with A20 cells, cells were first incubated with anti-mouse Fc (same as secondary/detection antibody but unlabeled) to block the Fc portion of IgGs expressed at the surface of A20 cells. The staining procedure was as detailed above. To assess antibody selectivity to human CD19 positive A20 cells, A20-hCD19-GFP cells were mixed with A20-mCherry cells at a 1:1 ratio and stained and analyzed using the protocol described above. Data are presented as the ratio between Mean Fluorescent Intensity (MFI) measured with A20-hCD19-GFP and A20-mCherry cells.

Antibody binding to red blood cells (RBC) in whole blood was determined as follows. First, test antibody was pre-incubated with a fluorescently-labeled Fab Fragment Anti-Mouse IgG (H + L) (115-007-003, Jackson ImmunoResearch, West Grove, PA, USA) at a 2:1 ratio for 15 min at 4 °C to minimize interference arising from the secondary (detection) antibody binding to cell surface and serum IgG. This antibody mixture was prepared 20 times concentrated and was added to heparinized blood from BALB/c mice and incubated for 15 min at room temperature. Blood cells were then washed three times and antibody binding was analyzed by flow cytometry. RBC were identified and gated based on their SSC-FSC parameters.

### 2.5. CD47 Competition Assay

A20-hCD19 cells were incubated with CD47-blocking test antibodies for 30 min at room temperature (rt). Test antibodies were then subject to competition by a fluorescently-conjugated low affinity anti-mouseCD47-blocking mAb (mAb3, see Figure S1) on ice for 20 min. After that, cells were washed and the percentage of test antibody outcompeted by the indicator antibody was measured by quantifying the MFI of cells by flow cytometry.

### 2.6. Phagocytosis Assay

A single cell suspension of bone marrow cells was obtained from BALB/c or C57BL/6J mice and cultured for 7 days in complete macrophage medium (RPMI 1640, 10% heat-inactivated FCS, 2 mM l-glutamine, 1 mM sodium pyruvate, 10 mM HEPES buffer, 50 μM 2-mercaptoethanol, 25 μg/mL gentamicin (Sigma-Aldrich, Buchs, Switzerland)) supplemented with 20 ng/mL M-CSF (PeproTech, London, UK). On the day of the experiment, non-adherent cells were eliminated by washing the culture flasks with PBS. Adherent macrophages were detached using a non-enzymatic cell dissociation solution (Sigma-Aldrich, Buchs, Switzerland) and washed in complete macrophage medium. For phagocytosis to proceed, macrophages were mixed with CFSE-labeled target cells (effector to target ratio of 1:5) in ultra-low attachment plates (Corning/Sigma-Aldrich, Buchs, Switzerland) and incubated in the presence of the test antibody for 2.5 h at 37 °C. Macrophages were subsequently stained with an Alexa Fluor 647-labeled anti-mouse anti-F4/80 secondary antibody (Clone A3-1, Biorad, Cressier, Switzerland) and a viability marker (Sytox Blue by Thermo Fischer Scientific, Reinach, Switzerland) was added to exclude dead cells before acquisition using a Cytoflex flow cytometer (Beckman Coulter, Indianapolis, IN, USA). Double-positive events (positive for F4/80 and CFSE-labeled target cell) were identified as phagocytosis events. Phagocytosis is presented as the percentage of macrophages which have engulfed at least one target cell (double-positive events/total macrophages × 100).

### 2.7. Isolation of HA-Specific TCR CD8^+^ T Cells

HA-specific TCR CD8^+^ T cells were isolated from lymph nodes and spleens of CL4 transgenic mice kindly provided by Dr. Roland Liblau (Research Center Toulouse Purpan, CPTP-INSERM). The CL4 mice were bred and maintained in Novimmune’s animal facility. Spleens and lymph nodes were digested using collagenase IV (Lubiosciences, Zürich, Switzerland) and DNase I (Sigma-Aldrich, Buchs, Switzerland) cocktail for 30 min at 37 °C. Single cell suspension was then processed through a negative CD8 isolation kit (STEMCELL Technologies, Vancouver, BC, Canada) following the manufacturer’s instructions. Purified CD8^+^ T cells were subsequently stained with CellTrace Violet (Thermo Fisher Scientific, Reinach, Switzerland) before being used in cross-presentation experiments.

### 2.8. Cross-Presentation Assay

Phagocytosis was set up as described above except for the incubation time at 37 °C, which was extended from 2.5 h to overnight. Then 4.5 × 10^4^ F4/80^+^ macrophages were sorted for each condition with an Astrios FACS sorter (Beckman Coulter, Indianapolis, IN, USA) and subsequently co-cultured with 17.5 × 10^5^ CellTrace Violet labeled, HA-specific TCR CD8^+^ T cells for 48 h at 37 °C. At the end of the macrophage/T cell co-culture, supernatants were collected and analyzed for IFN-γ and IL-2 levels using Luminex bead-based multiplex assay (Thermo Fisher Scientific, Reinach, Switzerland). In parallel, the proliferation of T cells stained with an anti-mouse CD8a antibody (Clone 53-6.7, BD Biosciences, San Jose, CA, USA) was evaluated by quantifying CellTrace violet proliferation peaks by flow cytometry.

### 2.9. In Vivo Efficacy Experiments

In vivo, 3 × 10^6^ A20-hCD19 cells were injected subcutaneously (sc) into the left flank of 6–8-week-old BALB/c mice (Charles River Laboratories). Tumors were measured every 2–3 days using a digital caliper and volumes were calculated using the formula (width × length × height × Pi)/6. Animals were euthanized when the tumor volume exceeded 1000 mm^3^ or at the experimental endpoint. In biAb monotherapy experiments, the treatment began when the tumors reached 6 × 6 mm. Mice were either treated intraperitoneally with 400 μg every other day or intratumorally with 100 μg of antibody every three days. The number of injections varied between experiments and is specified in the corresponding figure legend. In combination therapy experiments, mice were recruited when the tumors reached 100 mm^3^. For cyclophosphamide (CTX) combination therapy, we used a sequential combination therapy protocol as described by [12]. In brief, mice were treated with a single dose of 60 mg/kg CTX (Sandoz Pharmaceuticals AG, Rotkreuz, Switzerland) on day 6 followed by 100 μg antibody injected intratumorally on day 7, 10 and 13. For anti-PD-L1 combination therapy, mice received a mixture of 200 μg anti-PD-L1 (clone 10F.9G2, BioXcell, Lebanon, NH, USA) plus 400 μg biAb intraperitoneally on day 6, 8, 10, 12, 14. For rechallenge experiments, tumor-free mice and naive mice were injected (sc) with 3 × 10^6^ of either A20 wild type (wt) or A20-hCD19 cells on the opposite flank 2 months after rejecting the primary tumor. For B16F10-hCD19 experiments, 6–8-week-old C57BL/6J mice were engrafted with 1 × 10^5^ B16F10-hCD19 on the left flank and recruited once tumors became palpable. Mice received 1.2 mg antibody treatment on the day of recruitment and were subsequently treated every other day with 400 μg antibody.

### 2.10. Evaluation of biAb Bioavailability and Binding to RBC

BALB/c mice were injected intraperitoneally every other day with 400 μg antibody for a total of three injections. Mice were bled two days after the last injection to evaluate RBC concentration, antibody binding to RBC, and antibody bioavailability in the blood. RBC counts were determined using a HEMAVET 950 hematology analyzer (Drew Scientific Inc., Miami Lakes, FL, USA). To evaluate antibody binding to RBC, 2 μL of blood were washed three time with FACS buffer and subsequently stained with an anti-Mouse IgG (Life Technologies/Thermo Fisher Scientific, Reinach, Switzerland) for 20 min at room temperature. Cells were then washed three times in FACS Buffer and antibody binding was determined by flow cytometry.

For the assessment of blood bioavailability, blood was collected into serum separator tubes (BD Microtainer) and centrifuged at 10,000× *g* for 5 min. Resultant serum supernatant was stored at −20 °C until further analysis. Serum concentration of hCD19-specific antibody was determined by an ELISA, in which biotinylated hCD19 extracellular domain (produced in-house) was captured on streptavidin-coated plates. A dose range antibody spiked in serum from non-treated mice was used to calibrate the assay.

## 3. Results and Discussion

### 3.1. Generating Tumor Cells and Anti-CD47/TAA biAbs to Study Tumor-Specific CD47 Targeting In Vitro and In Vivo

To study immunosurveillance, and thus the ability to generate antitumor adaptive immune responses, we needed to work with immunocompetent mice and syngeneic tumor models. For the model tumor antigen, we chose to work with human CD19 (hCD19) transfected into an A20 cell line derived from a BALB/c diffuse large B cell lymphoma. In order to study biAb-mediated tumor-directed blockade of CD47, various anti-mouse CD47-targeting antibody sequences were identified using the κλ body biAb platform [36]. The two sequences analyzed in this study were initially expressed as mAbs and were shown to block mouse CD47-SIRPα interaction with different potency (CD47 mAb1 and CD47 mAb2, Figure S1). These sequences were then combined as biAb1 and biAb2 with the previously described high-affinity TAA sequence recognizing the human CD19 antigen, an anti-hCD19 sequence which is not cross-reactive with mouse CD19 [36]. Thus, biAb1 and biAb2 share the same anti-hCD19 arm but have different anti-mouse CD47 arms. These two anti-CD47 sequences bind overlapping epitopes on mouse CD47 but with significantly different affinities. The equilibrium dissociation constant (K_D_) between biAb1 and mouse CD47 is in the nanomolar range (Table S1), while the affinity of biAb2 to CD47 was too weak to be reliably measured. The CD47/CD19 biAbs and the corresponding monovalent CD47 and CD19 antibodies used in this study are schematically depicted in Figure S2.

### 3.2. CD47/CD19 biAbs Selectively Target Mouse Lymphoma Cells Expressing Human CD19

**Figure 1:**
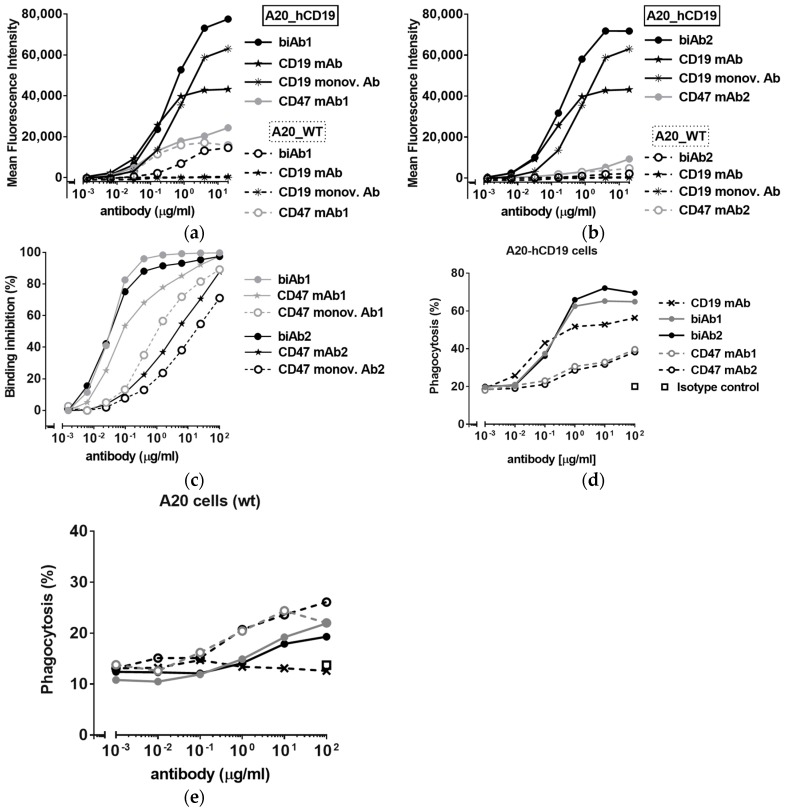
CD47/CD19 bispecific antibody (biAb) selectivity. (**a**,**b**) Binding, reported as mean fluorescence intensity values, to A20 mouse B cell lymphoma cells (A20 wild type (wt)) and A20 cells expressing human CD19 (A20-hCD19), of biAb1 (**a**) and biAb2 (**b**) as well as the anti-human CD19 mAb and the corresponding anti-mouse CD47 mAbs (mAb1 for biAb1 and mAb2 for biAb2). (**c**) Percent binding inhibition curves obtained by flow cytometry of a fluorescently-labeled CD47/ signal regulatory protein alpha (SIRPα) blocking mAb to A20-hCD19 cells by the biAbs and the corresponding anti-CD47 monovalent antibodies. (**d**,**e**) CD47/CD19 biAb-mediated antibody-dependent cellular phagocytosis (ADCP) as assessed by incubating fluorescently labeled A20-hCD19 (**d**) or A20 wt (**e**) cells with mouse macrophages (E:T ratio 1:5) in the presence of increasing concentrations of biAbs. Phagocytosis was assessed by flow cytometry and is expressed as the percentage of macrophages that ingested at least one target cell. The corresponding mAbs were tested for comparison.

After engineering, the A20-hCD19 cells displayed 30,000 copies of mouse CD47 per cell, similar to non-transfected A20 cells (“wild type”, A20 wt), and 65,000 copies of hCD19. Figure 1 shows that the targeting of A20-hCD19 tumor cells is primarily driven by the anti-hCD19 antibody arm of the biAbs. Both biAb1 and biAb2 bind strongly to A20-hCD19 cells, but only weakly (biAb1) or negligibly (biAb2) to A20 wt cells (Figure 1a,b). Of note, the binding of the two biAbs is comparable to CD19 monovalent antibody binding to A20-hCD19 cells, with a slight EC50 shift in favor of the biAbs, reflecting a contribution of the CD47 arms to the aggregate binding affinity (Figure 1a,b). To test if CD47/CD19 biAbs are able to block mouse CD47 on double-positive cells upon hCD19 co-engagement, we used a CD47 competitive binding assay. Both biAbs efficiently out-competed fluorescently labeled CD47 ligand (Figure 1c) more potently than the corresponding CD47 monovalent antibodies (i.e., κλ bodies with a non-binding arm instead of the anti-hCD19 arm) as well as the ‘bivalent’ CD47 mAbs. Noticeably, biAb1 and biAb2 blocked CD47 with similar potency despite different affinities of their anti-CD47 arms (see Table S1 and compare binding to A20 wt cells on Figure 1a,b) illustrating the power of avidity in facilitating CD47/TAA co-engagement at the cell surface.

### 3.3. Antitumor Efficacy of CD47-Blocking biAbs

The mouse IgG2a isotype binds activating Fcγ receptors with high affinity, mediating potent effector functions such as antibody-dependent cellular phagocytosis (ADCP). Phagocytosis of tumor cells can be further enhanced by blockade of CD47; the “don’t eat me” signal [3,4,7,8,9,37]. Here we show that biAb-mediated tumor-directed blockade of CD47 led to enhanced phagocytosis of A20-hCD19 cells in vitro as compared to anti-CD47 and anti-CD19 mAbs, anti-CD47 and anti-CD19 monovalent antibodies, or a combination of both monovalent antibodies (Figure 1d and Figure S3). We also show that the Fc region of CD47/CD19 biAbs is crucial for ADCP efficacy, as the F(ab’)2 fragments of biAb1 and biAb2 did not trigger the phagocytosis of target cells (Figure S4). As expected, the biAbs induced only minimal phagocytosis of hCD19-negative (A20-wt) cells (Figure 1e). As in the CD47 blocking assay (Figure 1c), biAb1 and biAb2 showed comparable activity in the ADCP assay (Figure 1d), confirming that: (i) the affinity for binding hCD19 is the driving force of biAb activity; and (ii) the avidity generated upon hCD19 co-engagement on the cell surface of double-positive cells forces efficient CD47 blockade, compensating for differences in CD47 arm affinity. It is also likely that CD47 binding and blockade on the tumor cell is locally stabilized at the site of the phagocytic synapse, arising through the interactions of antibody Fc regions with Fcγ receptors on the macrophage.

**Figure 2:**
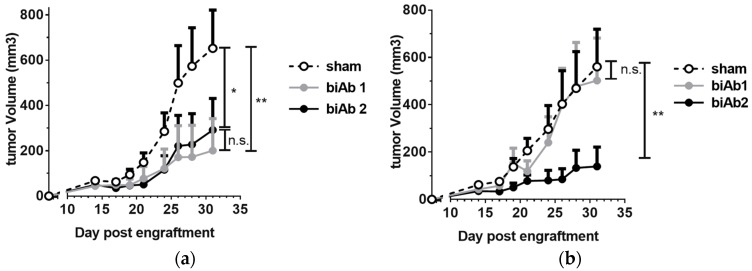
In vivo efficacy of CD47/CD19 bispecific antibodies. Treatment of BALB/c mice engrafted subcutaneously (sc) with A20-hCD19 cells began when the tumor size reached about 6 × 6 mm (*n* = 7 mice per group). (**a**) 100 μg of CD47/CD19 biAb was administered intra-tumorally on day 7, 10 and 13; (**b**) 400 μg of CD47/CD19 biAb was systemically administered intra-peritoneally on day 11, 13, 15 and 17. Tumor growth was measured three times a week and the average tumor volume per group +/− SEM (Standard Error of the Mean) reported. Statistical significance was determined using two way ANOVA comparing groups on d 31, *p*-value: * *p* < 0.05, ** *p* < 0.01; n.s., not significant.

To assess antitumor activity in vivo, BALB/c mice, engrafted subcutaneously (sc) with A20-hCD19 cells, received either intra-tumoral (it) or intra-peritoneal (ip) biAb treatment, initiated after the tumors reached a measurable volume. When administered locally, both biAbs were able to delay tumor growth (Figure 2a). However, upon ip administration, only biAb2 showed an antitumor effect (Figure 2b). This result was striking but not totally unexpected. While target cell selectivity of the CD47/CD19 biAbs is principally determined by the anti-hCD19 antibody arm, the higher-affinity anti-CD47 arm of biAb1 shows autonomous binding activity, in particular at higher antibody concentrations (Figure 1a). Thus, the higher affinity of the anti-CD47 arm in biAb1 resulted in a less stringent discrimination between double-positive and hCD19-negative cells as compared to biAb2 (Figure 1b). We hypothesized that the lack of in vivo efficacy of biAb1 administered systemically results from its interactions with CD47 expressed ubiquitously on healthy cells (i.e., the CD47 antigen sink), which, in turn, negatively impacts the pharmacokinetics, the level of toxicity, and eventually the efficacy of the biAb upon systemic administration.

### 3.4. The Affinity of the Anti-CD47 Arm Is Important for biAb Selectivity and Pharmacological Properties

**Figure 3:**
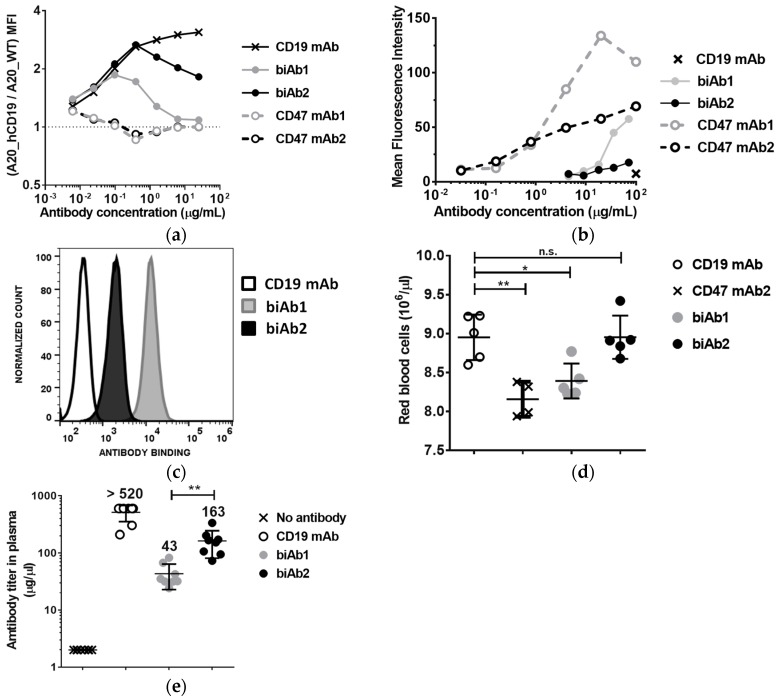
Affinity of the anti-CD47 arm influences biAb binding selectivity, hematological toxicity and bioavailability. (**a**) Binding selectivity was assessed using green fluorescent protein (GFP)-expressing A20-hCD19 cells and mCherry-expressing A20 wt cells at a 1:1 ratio, incubated with increasing concentrations of antibody and then analyzed by flow cytometry. The ratio between the Mean Fluorescence Intensity values obtained with A20-hCD19 cells and A20 wt cells is reported; (**b**) Antibody binding to erythrocytes from mouse (BALB/c) whole blood was determined by flow cytometry and the results reported as the Mean Fluorescence Intensity values obtained with biAbs and the corresponding mAbs; (**c**,**d**) To evaluate in vivo binding to RBC, BALB/c mice (*n* = 8/group) received three intraperitoneal 400 μg doses of anti-human CD19 antibody, biAb1 or biAb2 every 2 days. (**c**) Blood was collected 2 days after the last injection and RBC-bound antibody levels were determined by flow cytometry. Histogram plots show mean fluorescence intensity values obtained with the three antibodies tested; (**d**) Individual levels of RBC counts per mouse post antibody treatment; (**e**) Systemic bioavailability of the biAbs was assessed by obtaining the plasma and determining the antibody levels using a quantitative hCD19 ELISA assay. Normalized mean antibody titer +/− SD (standard deviation) is reported. Statistical significance was determined using one way ANOVA (**d**) or t-test (**e**); *p*-value * *p* < 0.05, ** *p* < 0.01; n.s., not significant.

To further assess the contribution of biAb anti-CD47 arms to cell binding, we incubated a 1:1 mix of fluorescently labeled A20-hCD19 cells and A20 wt cells with increasing concentrations of biAb and evaluated antibody binding by flow cytometry. Figure 3a shows relative binding, i.e., the ratio between MFI values obtained with A20-hCD19 and A20 wt cells. Contrary to the anti-CD47 mAbs, both biAbs showed a preference for double-positive cells (A20-hCD19), but biAb1 completely lost this binding selectivity at higher antibody concentrations (10 μg/mL or higher, Figure 3a).

We next assessed the binding of the CD47/CD19 biAbs to mouse red blood cells (RBC). RBCs displayed 20,000–25,000 copies of CD47 per cell. Given their abundance (between 6 and 10 billion cells per mL of blood), RBCs constitute potentially the most important antigen sink for anti-CD47 antibodies. To test binding to RBCs, antibodies were incubated with whole blood from BALB/c mice followed by flow cytometry analysis. BiAb1 bound mouse RBC relatively well at high concentrations, reaching binding levels comparable to anti-CD47 mAb2 at 100 μg/mL. In contrast, biAb2 showed only minimal binding (Figure 3b). Binding to RBC was also assessed in vivo two days after three ip injections of biAb at a dose of 20 mg/kg administered every other day. Consistent with the in vitro assay results, in vivo RBC binding levels were significantly higher with biAb1 than with biAb2 (Figure 3c). These results confirm once again the higher propensity of biAb1 to interact with the CD47 antigen sink. As anti-CD47 mAbs induce cytopenia and anemia in nonclinical and clinical studies, we assessed the effect of the two CD47/CD19 biAbs on RBC depletion. As shown on Figure 3d, RBCs declined following treatment with an anti-CD47 mAb (i.e., CD47 mAb2), to a lesser extent with biAb1, but, importantly, not with biAb2.

To assess how the ubiquitously expressed nature of CD47 affects biodistribution and pharmacokinetics of the biAbs, we determined the levels of circulating antibody two days after the last of three ip injections administered as described above. We observed a decreased bioavailability in the blood of both biAbs as compared to a non-binding control IgG (anti-hCD19 mAb) (Figure 3e). Consistent with the RBC binding results, biAb1 levels were significantly lower than biAb2 in circulation (Figure 3e).

These findings highlight the fact that the CD47 antigen sink could be a major challenge not only for monospecific anti-CD47 mAbs and SIRPα-Fc fusion proteins, but also for bispecific antibodies targeting CD47, depending on the affinity of the anti-CD47 arm. However, as illustrated by the biAb2, with a properly tuned affinity, negative effects of the CD47 antigen sink on bispecific drug pharmacology and safety can be minimized without compromising the efficacy of TAA-mediated, tumor-directed CD47 blockade.

### 3.5. CD47/CD19 biAb Induces Adaptive Antitumor Immunity

**Figure 4:**
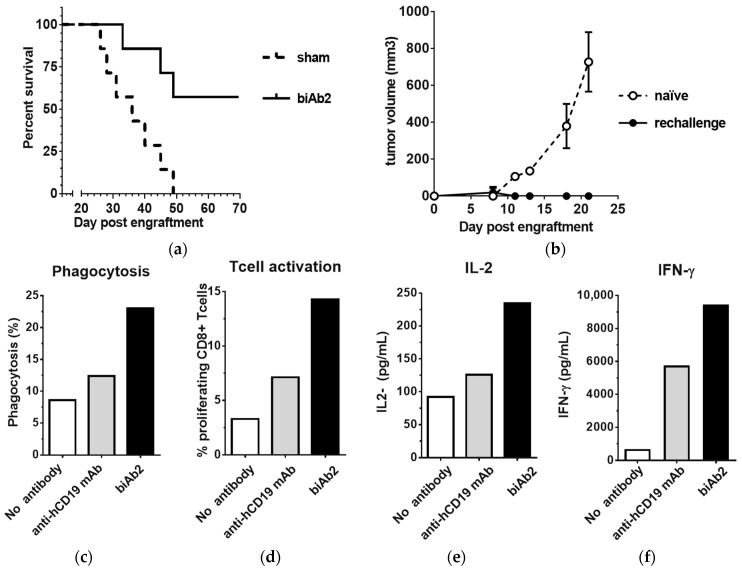
CD47/CD19 biAb promotes adaptive antitumor immunity. (**a**) Kaplan–Meier survival curves of animals treated with biAb2 in the experiment presented in Figure 2b; (**b**) Mice that rejected the A20-hCD19 primary tumor were rechallenged on the opposite flank with A20 wt cells and followed for tumor growth. The average tumor volume compared to naïve mice control group (*n* = 7) is reported. (**c**–**f**) biAb-mediated phagocytosis augments antigen cross-presentation and CD8^+^ T cell activation. Results from a representative experiment are presented; (**c**) Phagocytosis of HA-expressing tumor cells was assessed by incubating A20-hCD19-GFP-HA cells with bone marrow-derived macrophages from BALB/c mice and 5 μg/mL of antibody overnight. Phagocytosis was determined by flow cytometry and is expressed as the percentage of macrophages that ingested at least one target cell. The anti-human CD19 mAb was tested for comparison. (**d**) CD8^+^ T cell activation was assessed using macrophages from (**c**), co-cultured with cell tracer-labeled naive HA-specific TCR transgenic CD8^+^ T cells. The percentage of proliferating T cells was determined after 48 h of co-culture. (**e**,**f**) Cytokine secretion associated with T cell activation was assessed by quantifying the levels of IL-2 (**e**) and IFN-γ (**f**) released in the supernatant upon T cell activation.

As it has been reported that CD47-SIRPα blockade enhances the T cell-dependent antitumor response, we also studied the induction of adaptive immunity. Using our system, we observed that biAb2 administered systemically was not only able to slow-down tumor progression (Figure 2b) but also could provoke complete tumor regression in some animals and generate long-term survival (Figure 4a), the latter suggesting creation of efficient tumor immunosurveillance. We hypothesized that animals that rejected the primary hCD19-positive tumors may have developed immunological memory against A20 tumor antigens other than the xenogeneic neo-antigen, hCD19. To test if indeed the antitumor memory responses were associated with epitope spreading, mice with complete responses were rechallenged with wild type A20 cells. In contrast to the control group (i.e., not treated with biAb nor previously challenged with tumor), none of the previously treated and then rechallenged mice developed tumors (Figure 4b), indicating the induction of long-lasting protective antitumor immunity as well as a broadened antigen specificity.

To further define how tumor-directed blockade of CD47 resulted in enhanced adaptive immune responses, an in vitro cross-priming system using hemagglutinin A (HA) CD8^+^ transgenic T cells was established. The A20-hCD19 tumor cells were further transfected with HA (A20-hCD19-HA cells) to serve as the target tumor cell, and macrophages derived from mouse bone marrow (BMDMs) were serving as antigen-presenting cells. In the presence of biAb2, a substantially higher level of phagocytosis is observed as compared to the higher affinity anti-hCD19 mAb (Figure 4c). Furthermore, biAb2 promoted higher levels of T cell proliferation and cytokine production than with anti-hCD19 mAb-induced macrophages (Figure 4d–f). From these data, we conclude that enhanced tumor cell phagocytosis and antigen cross-presentation may contribute to in vivo efficacy of CD47-blocking biAbs. In any case, inhibition of CD47 on tumor cells promoted tumor control in vivo, as biAb2 demonstrated superior efficacy compared to the anti-hCD19 monovalent antibody and the anti-hCD19 mAb (Figure S5).

### 3.6. CD47/CD19 biAbs Enhance the Therapeutic Effect of Immune Checkpoint Blockade and Chemotherapy

**Figure 5:**
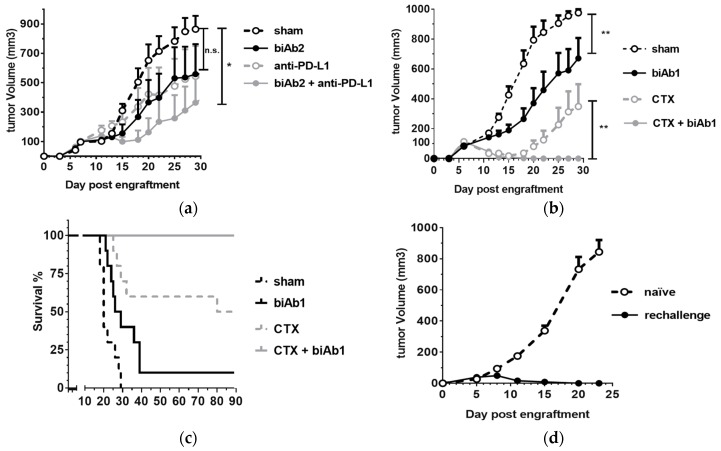
Combination therapies with anti-CD47 biAbs. (**a**) Combination of biAb2 and PD1/PD-L1 checkpoint blockade was assessed by engrafting BALB/c mice sc with A20-hCD19 cells and starting treatment when the tumor size reached 100 mm^3^ (intra-peritoneal doses of 400 μg for biAb2, or 200 μg doses for an anti-mouse PD-L1 antibody or a combination of the two antibodies on day 7, 9, 11, 13 and 15; *n* = 6 per group). (**b**,**c**) Combination of biAb2 and cyclophosphamide (CTX) was assessed by engrafting BALB/c mice sc with A20-hCD19 cells and starting treatment when the tumor size reached 100 mm^3^ (a single dose of CTX, 60 mg/kg ip, on d6, or three intra-tumoral 100 μg doses of biAb1 on day 7, 9 and 12, or a combination of both; *n* = 10 per group) (**b**) Tumor growth is shown as average tumor size per group +/− SEM. Statistical significance was determined using two-way ANOVA, *p*-value: * *p* < 0.05, ** *p* < 0.01; n.s., not significant; (**c**) Kaplan–Meier curves of tumor-bearing mice; (**d**) Mice that rejected the A20-hCD19 primary tumor were rechallenged on the opposite flank with A20-hCD19 cells and followed for tumor growth. The average tumor volume compared to naïve mice control group is reported.

PD-1/PD-L1 checkpoint inhibitors, while curative in some patients, have clinically demonstrated the multifactorial primary and/or acquired resistance mechanisms that tumors use to thwart immunosurveillance [38,39]. Thus, drug combinations aimed at overcoming these resistance factors, are an area of intense clinical research. As our results demonstrate the ability to enhance antigen presentation and endogenous T cell immunity, we hypothesized that tumor-directed blockade of CD47 with biAbs offer yet another means of attack. Indeed, CD47 blockers have been shown to increase tumor control when combined with anti-PD-1/PD-L1 antibodies in preclinical models [13,15,16] and CD47 expressed on tumor cells has recently been identified as a key agent of resistance to PD-1 immunotherapy in mice [20]. Thus, we next examined the efficacy of a combination therapy involving PD-1/PD-L1 checkpoint inhibition with a CD47-blocking biAb. We observed that while an anti-PD-L1 mAb or biAb2 single agent treatment was able to delay A20-hCD19 tumor growth, the combination therapy was more potent (Figure 5a).

We next studied the combination using cyclophosphamide (CTX). This chemotherapeutic agent promotes antitumor immunity by mediating selective immunodepletion of regulatory T cells [40]. We used a sequential combination therapy protocol to test the effect of tumor-directed blockade of CD47 in conjunction with CTX. A20-hCD19 tumors were allowed to grow to 100 mm^3^ and then the mice were treated with a single ip dose of CTX followed by three intra-tumoral injections of biAb1. The biAb, administered as monotherapy, was able to slow down tumor progression, whereas CTX led to a rapid tumor regression and durable responses in 50% of the animals (Figure 5b,c). Strikingly, the combination therapy allowed an even more potent effect, as 100% of the animals eliminated the tumor (Figure 5b,c) and developed long-term protective immunity, as demonstrated by resistance to tumor rechallenge (Figure 5d).

We used a second tumor type to confirm the antitumor efficacy of CD47-targeting biAbs. The B16F10 mouse melanoma cells that were stably transfected with human CD19 antigen (B16F10-hCD19 cells, Figure S6a) were engrafted sc in C57Bl/6 mice and treated intraperitoneally (ip) with biAb2. Similar to the results obtained with A20-hCD19 cells, we confirmed that binding of biAb2 to tumor cells is predominantly driven by its high-affinity anti-hCD19 arm (Figure S6b). We also demonstrated that tumor-directed blockade of CD47 with biAb2 administered systemically results in tumor growth inhibition and prolonged survival. (Figure S6c,d).

In conclusion, our study demonstrates that tumor-directed blockade of CD47 with dual targeting biAbs can inhibit tumor growth, promote tumor regression and induce durable antitumor memory responses. Our results further support the concept that CD47 blockade promotes T cell-dependent immunity via stimulating phagocytosis of tumor cells, antigen cross-presentation, and cytotoxic T cell cross-priming. We also show that tumor-directed blockage of CD47 can be combined with other therapeutic modalities, such as immune checkpoint inhibitors or chemotherapy, which should further improve antitumor responses and lift the proportion of curative therapies to offer to cancer patients. A key finding of this study is that using an in vivo system in which a CD47 antigen sink is present, the affinity of the anti-CD47 arm is important not only for drug safety and pharmacokinetics, but also for antitumor efficacy, in particular upon systemic administration. Therefore, the affinity of the CD47-blocking arm needs to be carefully considered when designing biAb antibodies targeting human CD47 for therapeutic intervention.

## Supplementary Materials

The following are available online at http://www.mdpi.com/2073-4468/7/1/3/s1, Figure S1: Blockade of CD47-SIRPα interaction with anti-mouse CD47 mAbs. Binding of fluorescently labeled recombinant mouse SIRPα to CD47 expressed on the surface of murine pre-B cell lymphoma L1.2 cells in the presence of increasing concentrations of anti-mouse CD47 mAbs was assessed by FMAT and is represented as Mean Fluorescence Intensity (MFI) ± S.D. of four replicates, Figure S2: Schematic representation of CD47/CD19 biAbs and the corresponding monovalent antibodies, Figure S3: biAb2-induced phagocytosis of A20-hCD19 cells, Figure S4: CD47/CD19 bispecific antibody Fc region is required for efficient ADCP, Figure S5: Comparison of in vivo efficacy of biAb2 and anti-CD19 monospecific antibodies, Figure S6. Antitumor efficacy of biAb2 in the B16F10-hCD19 model, Table S1: Affinity of biAb1 and biAb2 antibody arms.

Supplementary File 1
