# Supplementary material for: Tumor-Directed Blockade of CD47 with Bispecific Antibodies Induces Adaptive Antitumor Immunity
Source: Antibodies (Basel). 2018 Jan 3;7(1):3. doi: 10.3390/antib7010003 (PMC6698848; doi:10.3390/antib7010003)
Supplement: Supplementary File 1 [file antibodies-07-00003-s001.pdf]

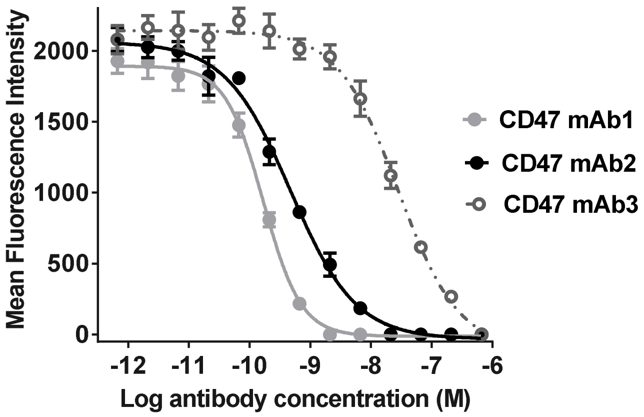

Figure S1. Blockade of CD47-SIRPα interaction with anti-mouse CD47 mAbs. Binding of fluorescently labeled recombinant mouse SIRPα to CD47 expressed on the surface of murine pre-B cell lymphoma L1.2 cells in the presence of increasing concentrations of anti-mouse CD47 mAbs was assessed by FMAT and is represented as Mean Fluorescence Intensity (MFI) ± S.D. of four replicates.

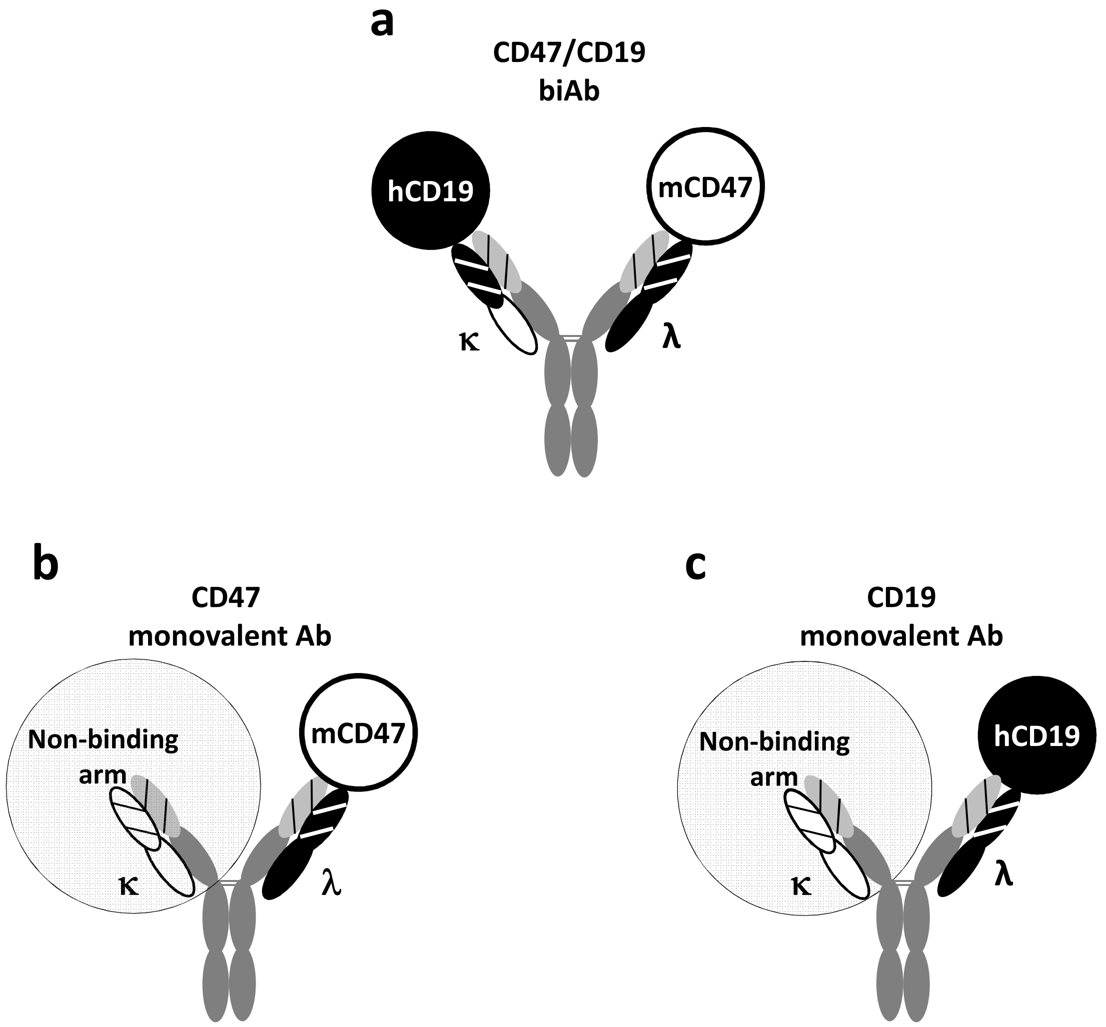

Figure S2. Schematic representation of CD47/CD19 biAbs and the corresponding monovalent antibodies. CD47/CD19 biAbs (**a**) and the monovalent Abs (**b,c**) are chimeric human-mouse κλ bodies [Fischer et al., *Nat. Commun*. 2015, 6, 6113]. Anti-mouse CD47 variable domains (**a,b**) anti-human CD19 variable domains (**a,c**) and variable domains from a neutral, non-binding mAb (**b,c**) were grafted on mouse heavy (IgG2a) and light chain constant domains. κ light chain, white; λ light chain, black; heavy chain, grey. VH and VL domains of human origin are marked with stripes.

a

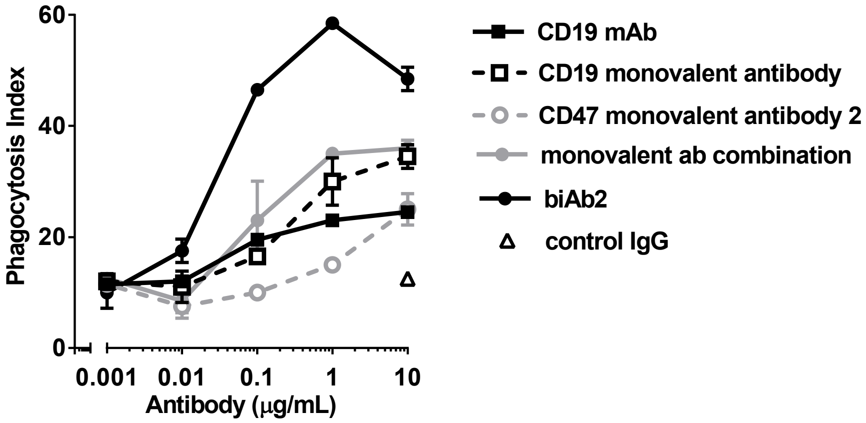

b

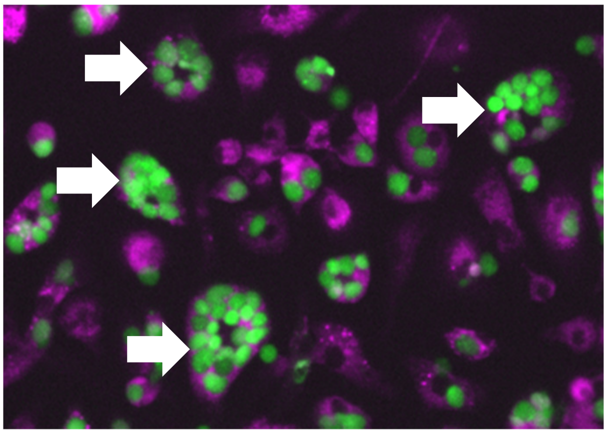

Figure S3. biAb2-induced phagocytosis of A20-hCD19 cells. **(a)** CD47/CD19 biAb mediated ADCP as assessed by incubating fluorescently labeled A20-hCD19 cells with adherent bone marrow derived mouse macrophages (E:T ratio 1:5) in the presence of increasing concentrations of biAbs. Phagocytosis was assessed by automated cellular quantitative fluorescent microscopy (CellInsight™ CX5 HCS Platform) and is expressed as the number of engulfed target cells per 100 macrophages (phagocytosis index). The corresponding CD19 and CD47 monovalent antibodies, or a 1:1 mixture thereof were tested for comparison. Results show the average of duplicates ± SD. **(b)** Example image showing phagocytosis of tumor cells (green) by macrophages (magenta) in the presence of biAb2. Arrows show macrophages that ingested more than 10 target cells.

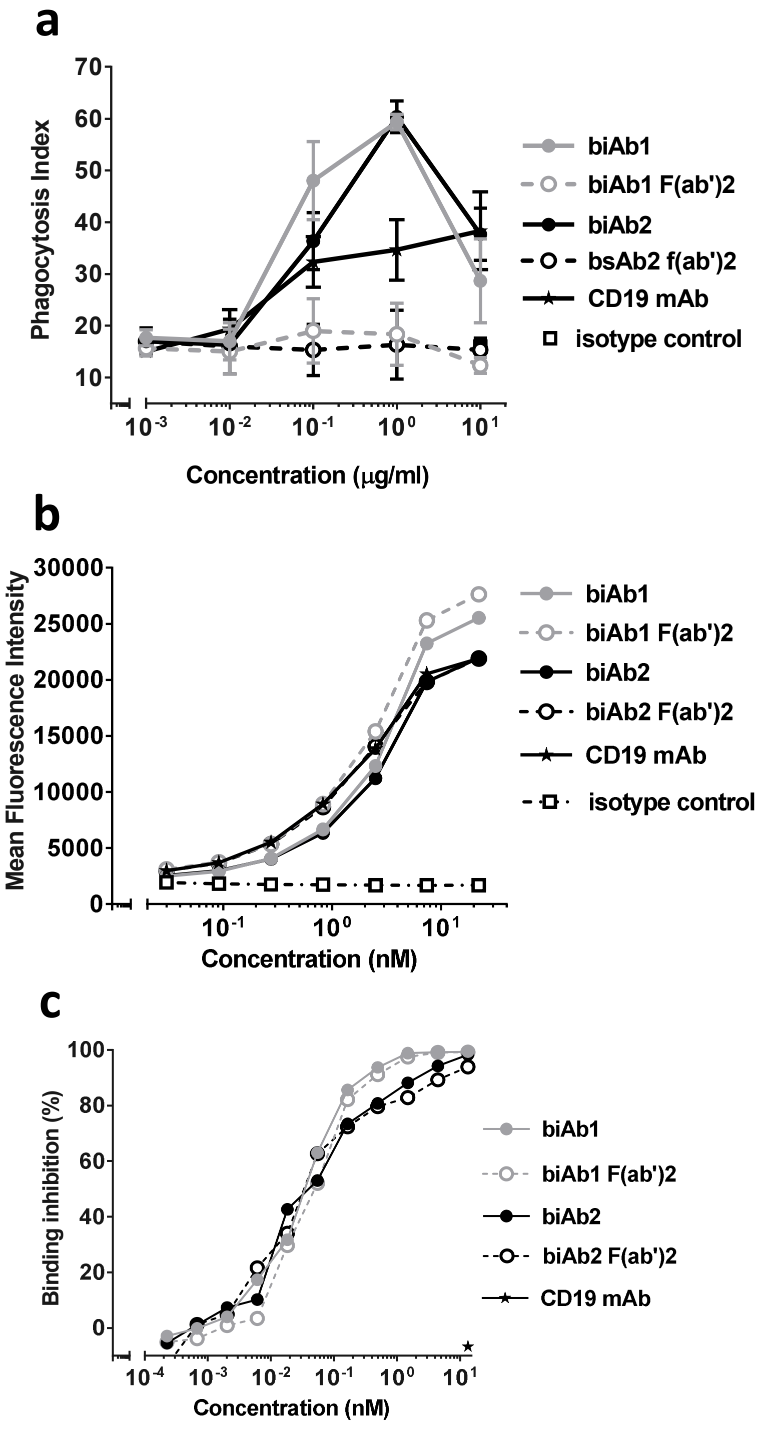

Figure S4. CD47/CD19 bispecific antibody Fc region is required for efficient ADCP. **(a)** Phagocytosis of A20-hCD19 cells mediated by biAb1, biAb2, or the F(ab')2 fragments thereof was assessed as described in Figure S3. The CD19 mAb was tested for comparison. Results show the average of quadruplicates ± SD. **(b)** Binding of biAbs (κλ), the corresponding F(ab')2 fragments and the CD19 mAb (λλ) to A20-hCD19 cells was tested by flow cytometry using an anti-mouse lambda light chain secondary antibody. **(c)** Blockade of CD47 on the surface of A20-hCD19 cells with biAbs and the F(ab')2 fragments. Binding of fluorescently labeled recombinant mouse SIRPα was measured with the CellInsight™ CX5 HCS Platform and is expressed as percent of binding inhibition.

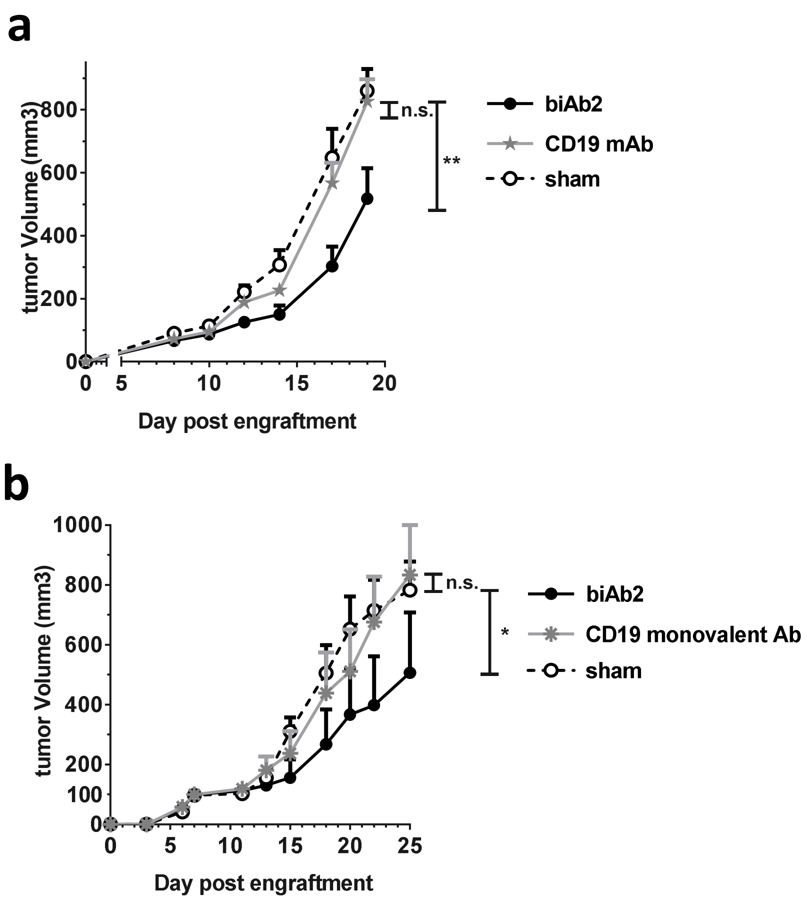

Figure S5. Comparison of *in vivo* efficacy of biAb2 and anti-CD19 monospecific antibodies. **(a)** Balb/c mice were engrafted sc into the upper left flank with $5x10^6$ A20-hCD19 cells. Treatment with of 400 mg intraperitoneal doses of biAb2 or the anti-hCD19 mAb was initiated on d6 and continued on d8, 10, 12, 14 and 16 (n=10 mice per group) **(b)** Balb/c mice engrafted as in (a) were treated with 400 mg intraperitoneal doses of biAb2 or the anti-hCD19 monovalent antibody on d7, 9, 11, 13, and 15 (n=6 mice per group). Tumor growth was measured three times a week and is shown as average tumor size per group +/- SEM. Statistical significance was determined using two-way ANOVA, p-value: * p<0.05, ** p<0.01; ns, not significant.

a

Ag copies/cell	B16F10	B16F10-hCD19
Mouse CD47	30’000	30’000
Human CD19	(---)	75’000

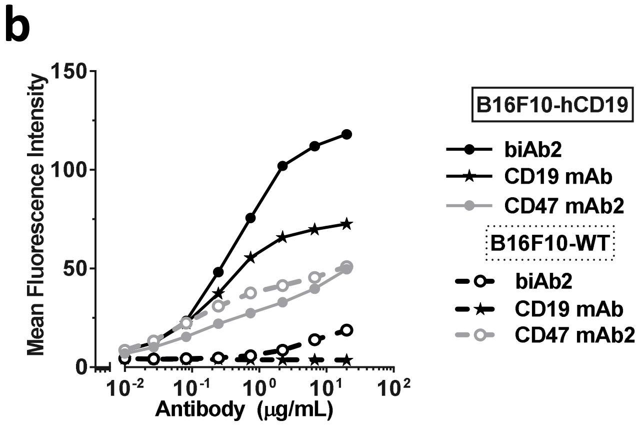

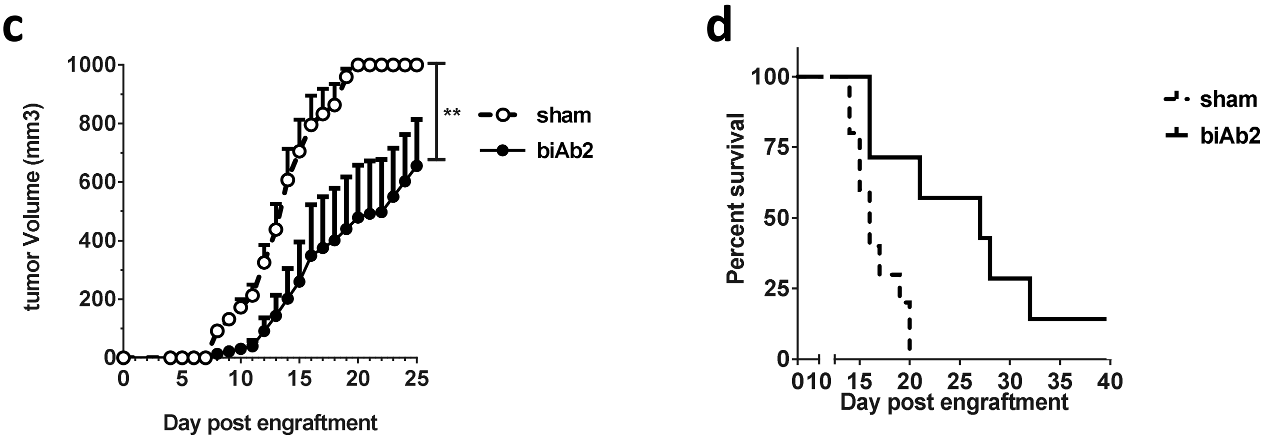

Figure S6. Antitumor efficacy of biAb2 in the B16F10-hCD19 model. B16F10 mouse melanoma cells stably transfected with human CD19 (B16F10-hCD19 cells) were used to assess the efficacy of anti-CD47/CD19 bispecific antibodies. **(a)** Mouse CD47 and human CD19 antigen cell surface density in B16F10 and B16F10-hCD19 cells. **(b)** Binding of biAb2 to B16F10 wt B16F10-hCD19 cells as assessed by flow cytometry, with the corresponding monoclonal antibodies tested for comparison. **(c, d)** *in vivo* efficacy of biAb2 in the B16F10-hCD19 model. C57BL6 mice were engrafted subcutaneously with B16F10-hCD19 cells and treatment began when tumors were palpable. Mice received 1.2 mg i.p. antibody treatment on the day of recruitment followed by 3x weekly i.p. injections of 400 µg antibody. **(c)** Tumor growth is shown as average tumor volume per group +/- SEM (n=10 mice per group). Statistical significance was determined using two-way ANOVA and is showing group comparison at day 25; p-value : ** p<0.01. **(d)** Kaplan-Meier survival curves of tumor-bearing mice.

Analyte	Target ag	K_D (nM)	ka (1/Ms)	kd (1/s)
anti-CD47 arm 1	mCD47	21.3	$2.2.10^{5}$	$4.7.10^{-4}$
anti-CD47 arm 2 *	mCD47	n.d.		
anti-CD19 arm	hCD19	0.6	n.a.	n.a.

Table S1. Affinity of biAb1 and biAb2 antibody arms. The affinity for recombinant mouse CD47 was determined using bio-layer interferometry (Octet®, Forte Bio). The affinity to native human CD19 expressed on Raji human B cell lymphoma cells was determined using Kinetic Exclusion Assay (KinExA®, Sapidyne). n.a., not applicable, n.d., not determined. * The affinity of the anti-CD47 antibody arm 2 was too weak to be measured reliably.

Supplementary Material and Methods

Affinity measurement

CD47 targeting arm affinity was measured on recombinant mouse CD47 protein using the Octet system (ForteBio). Streptavidin biosensors were loaded with 1 µg/mL biotinylated mouse CD47 (produced in house) for 10 minutes. After a 2 minute equilibration phase in Octet buffer (PBS, pH 7.4, 0.01% BSA, and 0.002% Tween 20), test antibody association kinetics was monitored for 10 minutes (at multiple antibody concentrations, ranging from 0.3 to10 µg/mL). Biosensors were subsequently soaked in fresh octet buffer for 10 minutes to determine antibody dissociation kinetics.CD19 targeting arm binding affinity was measured on human CD19 positive Raji cells using a KinExA instrument (Sapidyne) as described in [36]

CD47 competition assay with anti-mCD47 mAbs

L1.2 cells (a murine pre-B cell lymphoma cell line expressing ~60’000 CD47 molecules at the cell surface) were suspended in FACS buffer and dispersed into 384-well clear-bottom plates (Corning) at 3000 cells/well in the presence of increasing concentrations of CD47-blocking monoclonal antibodies. After an incubation of 10 min, a mixture containing soluble, histidine-tagged mouse SIRPα (#50956-M08H , Sino Biological) and detection antibody (anti HIS-biotinylated antibody (Qiagen) mixed to Streptavidin-Cy5 (Invitrogen)) was added to each well. After an incubation period of 2.5h, SIRPα binding to CD47 was determined using the FMAT 8200 Cellular Detection System (Fluorescent Microvolume Assay Technology, Applied Biosystems).

F(ab’)2 generation

F(ab’)2 fragments of biAb1 and biAb2 were generated using the FragIT Z microspin columns (Genovis) according to the manufacturer’s instructions. Removal of Fc fragments was achieved by exposing the cut IgGs (containing both F(ab’)2 and Fc fragments) to the CaptureSelect™ IgG-Fc (ms) Affinity Matrix (ThermoFisher Scientific). The purity of the resulting F(ab’2) fragments was assessed on a 2100 Bioanalyzer (Agilent).

Flow cytometry assay with F(ab')2 antibody fragments

A20-hCD19 cells pre-incubated with Mouse BD Fc Block™ (#553142, BD Pharmingen) for 15 min at 4°C were washed with FACS buffer and incubated with test antibodies or F(ab')2 fragments for 15 min at 4°C. Cells were washed once to remove unbound antibody and stained with a fluorescently labeled goat anti mouse lambda light chain polyclonal antibody (#1060-02, Southern Biotech) for 15 min at 4°C. A viability marker, SYTOX blue (#S34857, Thermo Fischer Scientific) was added before acquisition to exclude dead cells. Antibody binding was assessed by flow cytometry (Cytoflex®, Beckman Coulter) and FACS data were analyzed with FlowJo software (TreeStar). Dead cells were removed from the analysis.

Supplementary Material and Methods (cont)

CD47 competition assay with F(ab')2 antibody fragments
A20-hCD19 cells stained with 1µM of CFSE violet (#C34557, Thermo Fischer Scientific) were pre-incubated with Mouse BD Fc Block™ (#553142, BD Pharmingen) for 15 min at RT, washed once and incubated with test antibodies or F(ab')2 fragments for 50 min at RT. Cell-antibody mixtures were then dispersed into 384-well clear-bottom plates (#3764, Corning) at 3000 cells/well and incubated in the presence of a mix of mouse SIRPa-Fc (#50956-M02H , Sino Biological) and fluorescent anti-human Fc antibody (#109-606-170, Jackson Immuno Research) for additional 3 hours. SIRPα binding to CD47 was eventually determined using a CellInsight® CX5 imaging platform (Thermo Fisher Scientific).

Assessment of phagocytosis by automated high throughput quantitative microscopy
Bone marrow cells were extracted and processed as described in the Materials and Methods section. On day 5 of the macrophage differentiation protocol, macrophages were harvested and replated in complete medium in clear bottom black wall 96 well plates (Costar) at a density of 30 000 macrophages per well, and cultured for 2 additional days. For phagocytosis, adherent macrophages were labeled using calcein red-orange (Thermo Fisher Scientific) and co-incubated with 150’000 calcein AM (Thermo Fisher Scientific) -labeled target cells (effector to target ratio 1:5) in the presence of test antibody for 2.5 hours at 37°C. Phagocytosis data was acquired using a CellInsight™ CX5 High Content Screening Platform (Thermo Fisher Scientific) and analyzed using the HCS Studio™ Cell Analysis Software (Thermo Fisher Scientific). Phagocytosis index was calculated as the number of ingested target cells per 100 macrophages.
